# Evolution, immunity and the emergence of brain superautoantigens

**DOI:** 10.12688/f1000research.10950.1

**Published:** 2017-02-21

**Authors:** Serge Nataf

**Affiliations:** 1 Bank of Tissues and Cells, Lyon University Hospital (Hospices Civils de Lyon), CarMeN Laboratory, INSERM 1060, INRA 1397, INSA Lyon, Université Claude Bernard Lyon-1, Lyon, F-69000, France

**Keywords:** autoantigens, immune repertoire, natural autoantibodies, immunological homunculus, synapse, paraneoplastic syndrome, autoimmunity, central nervous system

## Abstract

While some autoimmune disorders remain extremely rare, others largely predominate the epidemiology of human autoimmunity. Notably, these include psoriasis, diabetes, vitiligo, thyroiditis, rheumatoid arthritis and multiple sclerosis. Thus, despite the quasi-infinite number of "self" antigens that could theoretically trigger autoimmune responses, only a limited set of antigens, referred here as superautoantigens, induce pathogenic adaptive responses. Several lines of evidence reviewed in this paper indicate that, irrespective of the targeted organ (e.g. thyroid, pancreas, joints, brain or skin), a significant proportion of superautoantigens are highly expressed in the synaptic compartment of the central nervous system (CNS). Such an observation applies notably for GAD65, AchR, ribonucleoproteins, heat shock proteins, collagen IV, laminin, tyrosine hydroxylase and the acetylcholinesterase domain of thyroglobulin. It is also argued that cognitive alterations have been described in a number of autoimmune disorders, including psoriasis, rheumatoid arthritis, lupus, Crohn's disease and autoimmune thyroiditis. Finally, the present paper points out that a great majority of the "incidental" autoimmune conditions notably triggered by neoplasms, vaccinations or microbial infections are targeting the synaptic or myelin compartments. On this basis, the concept of an immunological homunculus, proposed by Irun Cohen more than 25 years ago, is extended here in a model where physiological autoimmunity against brain superautoantigens confers both: i) a crucial evolutionary-determined advantage via cognition-promoting autoimmunity; and ii) a major evolutionary-determined vulnerability, leading to the emergence of autoimmune disorders in *Homo sapiens*. Moreover, in this theoretical framework, the so called co-development/co-evolution model, both the development (at the scale of an individual) and evolution (at the scale of species) of the antibody and T-cell repertoires are coupled to those of the neural repertoires (i.e. the distinct neuronal populations and synaptic circuits supporting cognitive and sensorimotor functions). Clinical implications and future experimental insights are also presented and discussed.

## Introduction

The role of auto-immune mechanisms in a large array of diseases continues to be extensively explored, and the identification of new target autoantigens is still an active field of research. However, despite the quasi-infinite number of potential target autoantigens that bear human cells, the majority of our internal antigenic library somehow remains off-target. Indeed, while many orphan autoimmune diseases have been described, the landscape of human autoimmunity is dominated by a limited number of disorders that include psoriasis, diabetes, vitiligo, thyroiditis, rheumatoid arthritis and multiple sclerosis. Moreover, besides "idiopathic" autoimmunity, for which no causative event can be conclusively identified, it is worth noting that "incidental" autoimmunity, triggered by neoplasms (paraneoplastic syndromes), vaccination (autoimmune/autoinflammatory syndrome induced by adjuvants [ASIA]) ^1^ or microbial infections (post-streptococcal glomerulonephritis and Guillain-Barre syndrome secondary to *Campylobacter jejuni* infection), does not affect all tissues and organs with an evenly distributed incidence. The great majority of such incidental autoimmune disorders clinically express as neurological pathologies and mainly target myelinic or neuronal autoantigens. Overall, these observations indicate that, independently from the MHC haplotype of an individual, autoantigens are not equal with regard to their potential for autoimmunity. There are what could be called superautoantigens, and in particular neural superautoantigens, toward which TCR and antibody repertoires tend to be skewed in humans.

It is proposed here that the existence of such superautoantigens is shaped by physiological events that are associated with human brain development and functions. The theory of the immunological homunculus, proposed more than 25 years ago by Irun Cohen ^2, 3^, constitutes an ideal framework to explain the emergence of superautoantigens during evolution. The present paper firstly provides a brief description of the somatosensory homunculus, i.e. the brain cortical area that, by analogy-based reasoning, inspired the concept of the immunological homunculus. Secondly, the immune and nervous systems are paralleled with regard to: i) the importance of self-generated inputs in the development of both somatosensory and immunological homunculi; and ii) the mechanisms driving a distorted representation of our body in both homunculi.

### The somatosensory homunculus provides a distorted representation of our body

**Figure 1.**
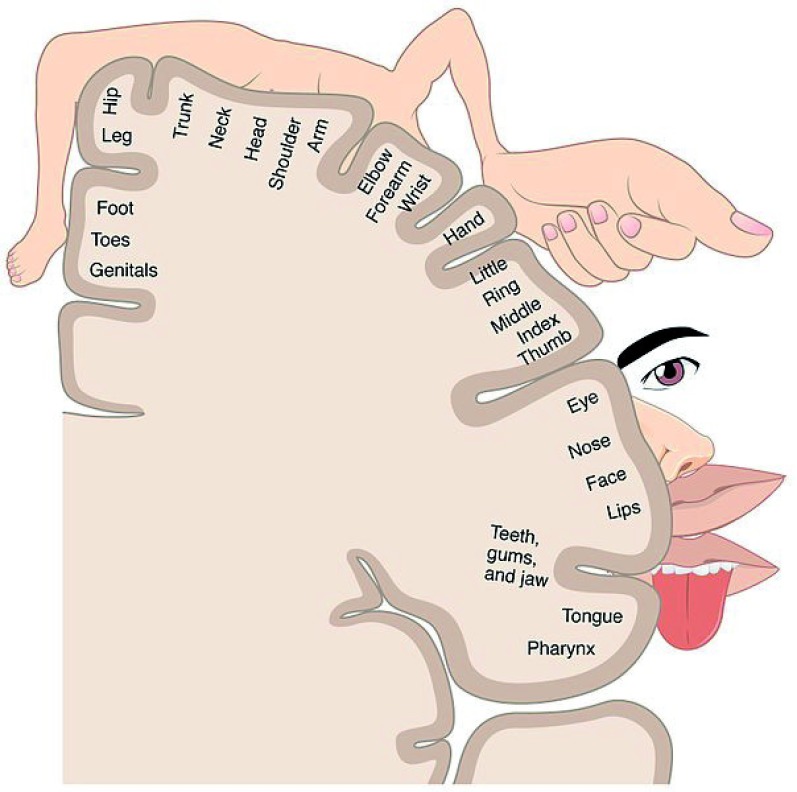
Representation of the somatosensory homunculus. The higher the density of sensory receptors in a given skin territory, the larger the surface covered by the corresponding cortical subarea that integrates inputs from this skin territory. In this anatomical and functional segmentation so-called somatotopy, the topographical heterogeneity of skin territories with regard to the density of sensory receptors is responsible for a distortion of our body representation in the sensory cortex. Thus, skin terminal nerves located in the thumb, lips or tongue are connected to a much larger brain cortical area than the terminal nerves innervating the whole trunk skin. The figure was obtained from Human Anatomy and Physiology, Chapter 14.2: Central Processing. OpenStax, Anatomy & Physiology. OpenStax CNX. Jul 30, 2014 ( http://cnx.org/contents/FPtK1zmh@6.27:KcreJ7oj@5/Central-Processing).

The somatosensory homunculus ( Figure 1) essentially relates to the sense of touch and neural connections that are established between i) innervated skin territories where peripheral receptors for touch sensory inputs are located and ii) specific subareas of the brain cortex where neurons that integrate touch sensory input are located. The higher the density of sensory receptors in a given skin territory, the larger the surface covered by the corresponding cortical subarea ^4, 5^. As a consequence, depending on their respective densities in sensory receptors, two skin territories covering quantitatively similar surfaces may be connected to cortical areas covering greatly different surfaces. In this anatomical and functional segmentation, so-called somatotopy, the topographical heterogeneity of skin territories with regard to the density of sensory receptors is responsible for a distortion of our body representation in the sensory cortex. For example, skin terminal nerves located in the thumb are connected to a much larger brain cortical area than the terminal nerves innervating the whole trunk skin ( Figure 1). From a functional point of view, this organization makes sense, since skin sensitivity needs to be highly efficient in anatomical territories requiring a finely tuned motor control, such as thumb, index, lips or tongue. Indeed, the acquisition of motor skills relies on bidirectional sensorimotor connections that allow motor and sensory activities to mutually fuel and integrate. The perception of our own motor activity, a process called sensory reafference (or sensory feedback), greatly participates in sculpting and refining motor programs ^6, 7^. Accordingly, in non-human primates, sensory loss in infancy profoundly alters the functional organization of the motor cortex ^8^. Conversely, specific motor programs that are, in part, evolutionary-determined, instruct the use-dependent development of specific subareas of the sensory cortex. Principally, this was shown in experiments where sensory reafferences driven by early primitive motor activity were found to model the sensory cortex of rodents ^9^. Finally, such feedback/feedforward processes between sensory and motor neuronal networks also operate in conditions of post-developmental motor learning ^10– 12^.

### The somatosensory homunculus is initially shaped by self-generated sensory inputs

For neuroscientists, the term "developmental plasticity" mainly refers to the generation of nascent neuronal networks, which during brain development recruit additional neurons and acquire a higher order of intra- and/or inter-network connectivity ^13, 14^. In this specific field of research, the visual cortex has offered a unique experimental paradigm to analyze the impact of sensory inputs on the development of sensory neuronal networks. Thus, in cats, rodents and non-human primates, deprivation of visual inputs during early life stages hampers the formation of a fully functional neuronal circuitry in the visual cortex ^15– 17^. In addition, recent studies performed in congenitally blind *vs* sighted humans demonstrated that both the functionality and connectivity of the visual cortex are, in part, shaped by visual experience ^18^. Finally, such an experience-dependent development of sensory neuronal networks was also demonstrated in the somatosensory cortex: trimming the whiskers of newborn rodents induces a partial deprivation of touch sensory inputs that is accompanied by profound developmental alterations of the somatosensory cortex ^19– 21^.

Importantly, it was demonstrated that sensory experience impacts on developmental plasticity during specific windows of time, so-called critical periods ^22, 23^, which vary depending on the sensory input considered. Moreover, besides the existence of critical periods defined by specific time frames of brain development, it is acknowledged that experience-dependent plasticity actually persists in the adult sensory cortices, although to a lower magnitude ^24, 25^. The most illustrative example is given by the cortical reorganization of somatosensory neurons that follows hand amputation in adults ^26, 27^.

Recent findings show that in addition to exogenous inputs, self-generated inputs shape the somatosensory homunculus. In particular, as mentioned earlier, our own motor actions, conscious or unconscious, are a constant source of self-generated sensory inputs that reinforce the sensory neural circuitry. Thus, twitches, especially frequent in newborns during sleep, trigger robust sensory reafference that shape the sensory cortex ^28^. Moreover, twitches are not randomly generated and have been proposed to provide a sensorimotor experience that helps build motor synergies for goal-directed wake movements, such as walking ^29, 30^. Lastly, that human fetuses perform synchronous and coordinated hand/mouth movements despite general motor immaturity ^31^ is thought to reflect an evolutionary-determined imprinting of such a motor behavior ^31^. In this regard, one may consider that nascent motor programs, which will ultimately support the achievement of species-specific motor behaviors (e.g. hand grasping, complex language-related oral motor skills and walking), provide a whole of self-generated sensory inputs that shape a distorted somatosensory homunculus. Overall, the distorted perception of our own body complementary relies on both experience-dependent and experience-independent (i.e innate) mechanisms that, in turn, are indispensable to the effective development and refinement of major human-specific motor programs.

### The immunological homunculus provides molecular support to physiological autoimmunity

The term "immunological homunculus" and the associated notion of physiological autoimmunity refers to the development of adaptive immune responses directed against self-generated inputs i.e. "self" antigens ^2, 3, 32^. Strikingly, human newborns harbor a non-maternally derived IgM repertoire that is directed toward autoantigens ^33– 35^. Given the sterile fetal environment, natural autoantibodies in newborns cannot result from mechanisms of cross-reactivity or molecular mimicry between "non-self" microbial antigens and "self" antigens. Interestingly, such a self-directed antibody repertoire has been proposed to form what could be called a "stem repertoire" from which networks of reactive and cross-reactive antibodies are progressively generated ^35– 38^. In the same manner, the frequency of T cell receptors (TCRs) recognizing autoantigens is much more than predicted by the clonal selection theory. Indeed, the negative selection of auto-reactive T-cells via AutoImmune REgulator gene (AIRE)-dependent epithelial expression of autoantigens is far from constituting a stringent process. An abundance of molecular and cellular interactions that do not relate to clonal deletion prevent physiologically-generated auto-reactive T-cells to exert pathogenic effects ^39– 41^. Moreover, in-depth analysis of public TCRs (i.e. TCRs that are shared by a large population of individuals in a given species) has shown that "self" peptides are frequently recognized by such public TCRs and could even be their main targets ^42, 43^. Thus, there is now compelling evidence that, as initially proposed by Irun Cohen, physiological autoimmunity does not only reflect incidental errors of the selection/tolerance immune machinery, but fulfills major functions under normal or pathological conditions. Notably, these include two major functions: i) anti-tumoral immune responses targeted toward developmentally-expressed autoantigens, which are re-expressed during the tumoral process ^33, 44^; and ii) support to cognition via the finely-tuned intra-central nervous system (CNS) activation of T-cells directed against brain antigens ^45– 48^. In addition, physiological autoimmunity against a specific set of "self" antigens may also prevent pathological autoimmunity against a distinct set of autoantigens. This was recently demonstrated in patients bearing AIRE mutations and exhibiting at the same time immune self-reactivity, responsible for pathological autoimmunity, and immune self-reactivity, protecting from pathological autoimmunity ^49^. Finally, another unexpected advantage conferred by physiological autoimmunity is to provide extended immune repertoires directed against "non-self" antigens. In point of fact, TCRs or antibodies directed against "self" antigens cross-react with a large range of "non-self" antigens, and physiological autoimmunity is essential for successfully tackling microbial infections. In particular, T-cell clones endowed with high reactivity against "self" antigens are major components of the adaptive immune response against infectious agents ^50– 52^. Thus, overall, the assumption that acquisition of an immunological homunculus represents a major educational step of the immune system development is now largely confirmed. 

### The immunological homunculus is shaped by a limited set of superautoantigens, toward which adaptive immune responses confer an evolutionary advantage

An important conclusion that needs to be drawn from the concept of immunological homunculus is that autoimmunity is by essence a physiological process that is required for the harmonious maintenance of our tissues and the fine adaptation of the human species to its environment. Accordingly, physiological autoimmune responses against superautoantigens should provide an evolutionary advantage to the human species. Indeed, when providing a distorted representation of our body, the somatosensory homunculus skews the focus of our perceptive competencies toward skin territories that are essential to the execution of major motor functions in humans, for example walking upright, hand grasping and speech. Similarly, one may propose that the immunological homunculus skews the focus of “self”-directed adaptive immunity toward a specific set of autoantigens that, in humans, represent functionally important targets of physiological autoimmunity. Logically, in humans and other species endowed with a developed neo-cortex (the brain area supporting cognitive functions, arising from the most recent evolutionary changes), brain-derived autoantigens should represent a major share of such a set of superautoantigens. Supporting this view, physiological mechanisms of cognition-promoting autoimmunity have been now extensively demonstrated in rodents. In particular, myelin-specific T-cell clones were shown to robustly stimulate neurogenesis *in vivo* via the synthesis of neurotrophic factors that are captured *in situ* by neural progenitors ^53, 54^. Conversely, T-cell deficient mice harbor profound cognitive alterations that can be reversed by adoptive transfer of CD4+ T-cells ^55, 56^. Interestingly, the cognition-promoting activity of T-cells was shown to specifically rely on a sub-population of memory T-cells recognizing brain-derived antigens and exhibiting homing properties toward meninges, choroid plexus and cervical lymph nodes (i.e. the regional lymph nodes draining cerebrospinal fluid) ^48, 57, 58^.

Most importantly, the notion of physiological autoimmunity suggests that pathological autoimmunity may not result from the *de novo* emergence of pathogenic autoreactive clones, but from pre-existing autoreactive clones that have acquired abnormal functional properties ^2, 44, 59^. In this regard, pathogenic autoimmunity and physiological autoimmunity should be expected to share the same preferential targets i.e. superautoantigens. Exposed below are several lines of evidence indicating that a major source of superautoantigens, targeted by both physiological and pathological autoimmunity, is provided by the CNS.

## The CNS is a major source of superautoantigens

### Antigenic compartments formed by myelin and synapses exhibit specific immunogenic properties

There are two categories of properties that confer a high immunogenic potential to myelin-derived and synapse-derived antigens:

1) *Abundance and high renewal rate*: While abundance (amount of antigen) is an important factor that determines our immune system's ability to see and react against an antigen, the renewal rate of an antigen is likely to be at least as important. In most cases, renewal implies degradation by the phagocytic system, which is an indispensable step to antigen presentation by mononuclear phagocytes. Conversely, a highly abundant antigen that is poorly renewed may be predicted to be poorly immunogenic. In this view, two categories of brain antigens fulfills the criteria of being both abundant and highly renewed: synapse-derived and myelin-derived antigens. Indeed, synapses are highly dynamic structures that are constantly submitted to a remodeling process supporting the generation and maintenance of operative and adapted neuronal networks. Neurons represent roughly half of all CNS cells and, depending on the brain area considered, each human neuron bears ~100,000 synaptic connections (as inferred from electron microscopy analyses of cortical samples) ^60^. In addition, the synaptic circuitry in humans is highly plastic until at least the third decade, which translates into a high rate of both *de novo* formation and elimination of synapses ^61^. Similarly, myelin (as assessed by measures of white matter volume) occupies nearly 25% of the total human brain volume ^62^ and was recently shown to be renewed at a very high rate in the steady state ^63^.

2) *Inflammation-associated development and function*: Microglial cells, brain-resident macrophages, play key regulatory functions during brain development and are currently considered as the main "architects" of nascent neuronal circuits ^64^. Such a unique function stands on the ability of microglia to exert finely tuned phagocytic activity and to synthesize a large range of cytokines, which not only control neuronal cell fate, but also the formation, selection, maintenance and remodeling of interneuronal synapses. Thus, during brain development, microglia successively engage distinct activation programs that are in close synchrony with the stepwise establishment and maturation of neuronal circuits ^65^. Moreover, in the mature brain, microglia constantly operate a complement-dependent phagocytosis of poorly-active synapses, thus preventing inappropriate connectivity. Lastly, several lines of evidence indicate that specific inflammatory cytokines regulate synaptic activity and function under physiological conditions ^66^. TNF-alpha secreted by glial cells preserves the efficacy of excitatory synapses ^67^, and IFN-γ is a key molecular support for excitatory synapses ^68^ and neuronal circuitry involved in social behavior ^69^. With regard to the myelin compartment, it is also worth noting that myelination of axons is a highly dynamic process that is coupled to synaptic activity ^70– 74^, and thus indirectly linked to physiological inflammation. In addition, microglia exert direct effects on the development and myelinating functions of oligodendrocytes (the myelin-forming cells) via the synthesis of H-ferritin ^75^ and M2-type cytokines ^76^. Overall, the formation, maintenance and plasticity of two major brain molecular compartments, namely myelin and interneuronal synapses, involve a set of exquisitely-controlled immune mechanisms. In this regard, physiological inflammation appears to be required to ensure proper CNS functions ^66^. In addition, while inflammatory mechanisms are now recognized in shaping brain development in rodents and humans, a major distinctive feature of the human brain is the duration of its development over a period of time that extends from early embryonic stages until adolescence ^77^ and beyond ^78^. Indeed, the acquisition of a fully-operative neuronal circuitry supporting primary human-specific brain skills (regarding emotional, cognitive and sensory-motor functions) is a decade-long process that is intimately associated to myelination ^78^. Along this line, the proliferation of neuronal progenitors and their differentiation into cortical neurons, usually designated by the term "corticogenesis", was shown to be much slower and complex in humans compared to rodents ^79^. Thus, besides development and maturation, adult synaptic plasticity, allowing the constant remodeling of synaptic connections in order to maintain, extend and/or reorganize neuronal circuits, is likely responsible for a massive exposure of the immune system to synapse- and myelin-related antigens throughout life. The recent demonstration of a rich lymphatic vasculature, which drains brain antigens to cervical lymph nodes ^80, 81^, further supports this view.

### Non-CNS autoimmune disorders target CNS antigens

While CNS autoimmune diseases are generally relatively infrequent, many autoantigens identified in non-CNS autoimmune pathologies are enriched in the synaptic fraction of the developing and/or mature brain. Below is a non-exhaustive list of such autoantigens:

1) *GAD65*: Glutamate decarboxylase 65 (GAD2), considered the main autoantigen in diabetes type I ^82^, is a synaptic enzyme that catalyzes γ-aminobutyric acid (GABA) synthesis from glutamate. Its synaptic expression in inhibitory terminals (i.e. axon terminals from neurons transmitting inhibitory inputs) is indispensable to the effective functioning of the GABAergic system (all neurons for which the primary neurotransmitter is GABA) ^83^.

2) *AchR*: Myasthenia gravis is mediated by autoantibodies targeting the AchR (acetylcholine receptor) on the post-synaptic membrane of the neuromuscular junction ^84^. However, acetycholine is also a key neurotransmitter in the CNS, and AchR-mediated synaptic transmission is essential in crucial cognitive functions, such as memory ^85^.

3) *HSPA5 and other heat shock proteins*: The human stress protein, HSPA5 (also known as BIP or GRP78), belonging to the heat shock protein family A (HSP70), is one of the autoantigens involved in the pathophysiology of rheumatoid arthritis ^86, 87^. It is also a major component of the synaptic glutamate receptor complex ^88^. Similarly, the heat shock protein HSP60, a predominant target of physiological autoimmunity ^89^, is abundantly expressed in axon terminals ^90^, and mutations in the HSP60 gene result in a human disorder affecting motor neurons (autosomal recessive spastic paraplegia 13) ^91^.

4) *Small nuclear ribonucleoproteins*: Small nuclear ribonucleoproteins (snRNPs) are core components of the spliceosome machinery and the main autoantigens toward which anti-ribonucleotide antibodies are directed in systemic lupus erythematous (SLE) and Sjögren’s syndrome ^92^. In neurons, specific families of mRNAs are exported toward axon terminals and synapses in structures called RNA granules or ribonucleoprotein particles. Such structures are essential to the proper trafficking of specific mRNA species at distance from the soma and their local translation in the synaptic compartment ^93^. Mutations or deletions in genes coding for RNA-binding proteins (RBPs) are involved in numerous inherited CNS disorders ^94^, including fragile-X mental retardation ^95^, spinal muscular atrophy and spinocerebellar ataxia ^96^, as well as familial forms of the following neurological conditions: autism ^97, 98^; amyotrophic lateral sclerosis ^99^; and fronto-temporal lobar degeneration ^100^. The whole spectrum of RNAs and proteins that are complexed to such neuronally-expressed RBPs is currently extensively explored by systemic approaches ^94, 101^ and include several snRNPs targeted by autoantibodies in SLE or Sjögren’s syndrome. These snRNPS comprise of the La autoantigen Ssb, which binds FRMP (fragile X mental retardation protein) ^101^, the U1 snRNP, which interacts with SMN (survival of motor neurons) ^102^, and the small non-coding RNA called Y RNA, a component of Ro60 ribonucleoprotein particle, which binds neuronal ELAV-like protein ^103^. Finally, ribosomal proteins are themselves targeted by both pathological autoimmunity in SLE patients ^104^ and physiological autoimmunity in healthy individuals ^105^. Again, synapses are specifically enriched in ribosomes and allow crucial synaptic proteins to be synthesized in a timely manner ^106, 107^.

5) *Basement membrane proteins*: Autoimmunity against collagen IV and laminins, two major components of basement membranes, is responsible for the development of anti-glomerular basement membrane glomerulonephritis, Goodpasture’s disease ^108^ and several autoimmune skin disorders ^109^. Recent evidence indicates that synapses are embedded in a extracellular matrix microenvironment, in which collagen IV and laminins are not only abundant, but critically involved in synapse morphogenesis and synaptic remodeling ^110– 113^.

6) *Tyrosine hydroxylase*: Vitiligo is a frequent autoimmune disease characterized by an immune-mediated destruction of melanocytes leading to skin depigmentation ^114^. Interestingly, the biochemical pathways allowing the synthesis of melanin and dopamine respectively present major similarities ^115, 116^, and the intra-CNS grafting of melanocytes was recently proposed as a therapeutic approach for Parkinson's disease, a neurodegenerative disorder affecting dopaminergic neurons ^115^. In this regard, tyrosine hydroxylase, a major enzyme of the dopamine synthesis pathway in neurons is also essential to melanin synthesis and is targeted by autoantibodies in vitiligo ^117, 118^. Also, the melanin-concentrating hormone receptor 1 (MCHR1), another identified autoantigen in vitiligo ^119^, is expressed by a subpopulation of CNS neurons and its ligand, MCH, is indeed a neuropeptide regulating energy balance, sleep and mood ^120, 121^.

7) *Thyroglobulin and acetylcholinesterase*: Thyroglobulin (TG) and thyroid peroxidase (TPO) are the two main thyroid autoantigens targeted in Hashimoto's disease ^122^. While anti-TPO antibodies have been shown to bind a subpopulation of astrocytes ^123^, it is worth noting that anti-TG autoantibodies recognize an acetylcholinesterase domain that is essential to both the immunogenicity of TG ^124– 126^ and its function ^127, 128^. Thus, cross-reactivity between TG and acetylcholinesterase, a target autoantigen in Myasthenia gravis, was proposed as a mechanism of ocular muscle dysfunction in Hashimoto's disease ^124^. As mentioned earlier, the cholinergic system, essentially supported by functional interactions between acetylcholine, acetylcholinesterase and AchR, is crucial to the execution of major cognitive tasks, such as learning and memory.

### Cognitive alterations are frequently observed in patients suffering from non-CNS autoimmune diseases

Subclinical cognitive alterations, as well as psychiatric symptoms, are observed in a large array of non-CNS autoimmune diseases. Interestingly, specific antibody signatures have been shown to be associated with such neurological or psychiatric manifestations, which argues against a non-specific inflammatory process that would essentially involve innate immune mechanisms. Below is a list of the main non-CNS autoimmune disorders that may associate with cognitive and/or psychiatric symptoms:

1) *SLE and Sjögren’s syndrome*: Besides purely neuropsychiatric forms of SLE, subtle to major cognitive alterations were demonstrated in up to 20% of SLE patients ^129^. Cognitive clinical signs in SLE are accompanied with high titers of anti-N-methyl-D-aspartate receptor (NMDA; also named NR2 glutamate receptor) and/or anti-ribosomal antibodies ^130, 131^. Similarly, cognitive dysfunctions along with brain structural alterations, detectable by magnetic resonance imaging (MRI), were reported in up to 65% patients suffering from primary Sjögren’s syndrome ^132– 134^. As in SLE patients, a correlation was observed between titers of anti-NR2 antibodies (in the serum or cerebrospinal fluid) and clinical sores of cognitive dysfunction ^130, 135^.

2) *Hashimto's thyroiditis*: Hashimoto's encephalopathy, also known as steroid-responsive encephalopathy associated with autoimmune thyroiditis (SREAT), is a rare condition in which anti-TPO antibodies are involved ^136^. However, apart from SREAT, autoimmune thyroiditis (AIT) patients who are in an euthyroid state, suffer from mild to severe cognitive alterations correlating with serum levels of anti-thyroid antibodies, in particular anti-TPO and anti- TG antibodies ^137, 138^.

3) *Rheumatoid arthritis*: While the rate of motor or sensory neurological symptoms is relatively low in rheumatoid arthritis (RA) patients, the incidence of mood disorders is estimated to reach up to 70% ^139^. Moreover, in independent studies, mild cognitive deficits were demonstrated in more than 70% of RA patients and were associated with MRI or biological signs of altered CNS tissue integrity ^140, 141^.

4) *Psoriasis*: Psoriasis is a chronic skin disorder that may also target joints, and for which several candidate autoantigens have been identified ^142^, including, surprisingly, the melanocytic autoantigen ADMTSL5 ^143^. The impact of psoriasis plaques on self-esteem and mood is well-described and the role of psychological stress as a trigger of psoriasis recurrence is also robustly documented ^144^. However, measurable signs of subtle cognitive impairment are also observed in psoriasis patients ^145, 146^, even during the early phases of the disease ^145^.

5) *Crohn's disease*: Inflammatory bowel diseases, including Crohn's disease (CD), associate with distinct profiles of circulating autoantibodies directed notably against glycan, GP2 and GM-CSF ^147^. While glycans were shown to be specifically enriched in synapses ^148, 149^, the intra-CNS expression of GP2 and GM-CSF in the developing or mature brain is still lacking. However, two recent MRI studies performed in CD patients demonstrated marked structural brain alterations ^150, 151^ that correlated with cognitive dysfunction ^150^.

### Myelin and synapses are by far the most frequently targeted compartments in CNS autoimmune disorders

 Besides multiple sclerosis, during which one or several myelin autoantigens are targeted ^152^, a flurry of rare CNS autoimmune disorders, notably including paraneoplastic syndromes (PNS), have been characterized in the past decade. Interestingly, not only do a great majority of PNS have a purely neurological expression, but autoantigens in PNS were found to derive essentially from the synaptic compartment ^153^. Moreover, other CNS autoimmune disorders not associated with neoplasms also target synaptic proteins, and the term "autoimmune synaptopathies" has been proposed to designate such pathologies ^153^. The following is a non-exhaustive list of the synaptic autoantigens currently identified: GAD65 (glutamic acid decarboxylase) ^154^; NMDAR (N-methyl-D-aspartate receptor) ^155^; AMPAR (α-amino-3-hydrozy-5-methyl-4-isoxazolepropionic acid receptor) ^156^; Caspr2 (contactin-associated protein-like 2) ^157^; LGI-1 (leucine-rich glioma-inactivated protein 1) ^158^; GABA-B receptor (γ-aminobutyric acid receptor B) ^159^; GABA-A receptor (γ-aminobutyric acid receptor A) ^160^; mGluR5 (metabotropic glutamate receptor 5) ^161^; GlyR (glycine receptor) ^162^; NRXN3 (neurexin-3α) ^163^; AMPH (amphiphysin) ^164^.

## On the "co-evolution" of the immune and nervous systems

As shown above, a high number of antigens targeted in CNS or non-CNS autoimmune diseases belong either to the myelin or synaptic compartments. Even though such target autoantigens are evidently also expressed in non-CNS locations, the important questions arising from such an observation are why and how the human immune repertoires are skewed toward brain superautoantigens. As discussed earlier, both anti-tumoral immunity and maintenance of tissue integrity are essential functions that can be assigned to physiological autoimmunity ^59, 165^. Nevertheless, these functions do not appear to afford an evolutionary advantage to humans over other species endowed with an adaptive immune system. One may consider that the human species is indeed essentially characterized by a particular ability to operate complex cognitive tasks and perform exquisitely precise motor programs. On this basis, it can be hypothesized that CNS-derived autoantigens are major targets of physiological immunity in humans. Moreover, at the scale of evolution, physiological autoimmunity against CNS auto-antigens may reflect not only the development of fine cognitive and motor functions in a given species, but the extent to which support to these functions is afforded by adaptive immunity in this species.

### From symbiosis to phylosymbiosis

Interactions between humans and their gut microbiota is an illustrative example of what could be termed an immune-mediated symbiotic relationship. On one hand, gut microbiota constantly stimulate the adaptive immune system and shape T-cell and antibody repertoires, thus expanding, through cross-reactivity, the diversity of adaptive immune responses. In turn, the adaptive immune system tightly controls the composition of gut microbiota and favors the development and maintenance of a long-lasting commensal flora, which is benefitial to the host. Immune response against gut microbiota fluctuates over time and serum antibody titers against microbiota-derived antigens are submitted to variations, determined by epitope-specific clonal expansion and dominance ^166^. Thus, more generally, the commensal flora residing in the skin, gut, lungs and urogenital tract permanently stimulates, shapes and modulates our whole immune repertoire ^167– 169^. In this state of "immunity by equilibrium" ^169^, populations of Tregs, deriving either from the thymus or peripherally-generated, play a crucial role in the neonatal development of tissue-specific tolerance toward symbiotic flora components ^170– 174^. While symbiosis corresponds to a process of co-development and mutual support between species, co-evolution is defined by mutual selective pressure exerted by two species to the benefits of both species. Interestingly, the term phylosymbiosis was recently proposed to depict the parallel demonstrated between the phylogeny of host-associated microbial communities and the phylogeny of species hosting these distinct communities ^175^. Of note, phylosymbiosis appears to be essentially mediated by the immune system of the host ^175^. In this regard, host/microbiota symbiotic interactions could be considered as resulting from a process of immune-mediated co-evolution of organisms.

### From co-development to co-evolution

By analogy with the notions of phylosymbiosis and co-evolution, it is proposed below that the immune and nervous systems not only co-develop at the scale of an individual, but have co-developed during evolution. This idea essentially stems from the crucial demonstration that human newborns harbor an IgM antibody repertoire directed against autoantigens ^34, 35, 38^. Obviously, and as previously mentioned, since the amnion forms a sterile environment, cross-reactivity against microbiota-derived antigens cannot explain such an observation. Interestingly, while the full array of autoantigens targeted by IgM in newborns remains to be identified, many of the currently known targeted autoantigens are components of the myelin or synaptic compartments. These include: GAD65, MOG and acetylcholinesterase (cf supra); HSP60, a mitochondrial chaperonin whose genetically-determined alterations lead to a familial form of motor neuron disease ^176^ (cf supra); myosin, a protein whose brain isoform is abundantly expressed in synapses ^177, 178^; galectin-1 and -3, two neuronally expressed molecules that bind to the synaptic RNA-binding protein SMN (Survival Motor Neuron) ^179, 180^; B2-microglobulin, a key immune molecule also required for proper CNS development and plasticity ^181^.

These observations suggest that the developing CNS provides a highly diverse array of autoantigens that may stimulate and somehow educate effector T and B cells during the prenatal period. The main advantage conferred to the host by such an educational process would be a pre-natal expansion of memory lymphocytes, which, through cross-reactivity, would provide a larger immune coverage against infectious agents (including pathological microbiota).

Beyond development, it is also suggested that throughout the lifetime of an individual, brain-derived autoantigens may constantly shape the repertoire of memory lymphocytes and provide tonic signals for the survival and self-renewal of naïve lymphocytes ^182– 184^. As shown above, synaptic remodeling and myelin renewal are two major hallmarks of physiological neural functions during the life span of an individual. In particular, the learning-mediated establishment of new neuronal circuits, their selection and maintenance or elimination implies a constant adjustment of our neural repertoires (neural repertoires being defined here as all neuronal populations and synaptic circuits available for cognitive or sensorimotor tasks at a given time). Not only will the brain continue to develop and maturate until early adulthood, but cognitive and sensori-motor functions in the mature CNS are inherently-linked to the plasticity of synapses and myelin sheaths. Overall, one may propose that, similarly to microbiota, the CNS constantly fuels the immune system with antigens that shape and modulate our T- and B-cell repertoires. Conversely, the CNS-instructed diversification of our immune repertoires may ensure that essential synaptic circuits are reinforced by cognition-promoting autoreactive lymphocytes. Thus, at the scale of an individual, the acquisition and maintenance of the immune and neural repertoires may be somehow coupled via a process of mutual development and support.

What about evolution? It is proposed here that such a coupling of immune and neural repertoires may have been a driving force of evolution. In this theoretical model, the nervous and immune systems would have been submitted to a co-evolution-like process, consisting of a mutual selective pressure exerted by both systems to the benefits of the host. On one hand, through cognition-promoting immunity, the evolutionary-determined emergence and diversification of adaptive immunity would have provided support to new neural repertoires. At the same time, the evolutionary-determined diversification of neural repertoires would have promoted new immune repertoires (and a subsequent larger ability to tackle infections) via exposure to a larger array of CNS-derived antigens. Accordingly, it may be predicted that among species endowed with an adaptive immune system: i) Diversity of the germline-encoded and realized (mature) immune repertoires parallels the diversity exhibited by the neural repertoire; ii) cognition-promoting autoimmunity is quantitatively and qualitatively scaled to the level of complexity that each species exhibits with regard to cognitive functions; and iii) genes involved in the diversification of both immune and neural repertoires have had a major evolutionary impact. Importantly, this model would explain why the realized human TCR repertoire overcomes the one of rodents by a factor of 10 ^185^.

### Critical points and limitations of the co-development/co-evolution model

*Co-development and co-evolution processes may also link the immune system to non-CNS organs:* The theoretical model discussed above may be considered as neurocentric. Indeed, the concept of protective autoimmunity likely applies also to non-CNS organs, and the CNS is not the only source of superautoantigens. Thus, in addition to synaptic and myelin antigens, other families of autoantigens are: i) highly renewed, ii) abundantly exposed to the immune system, iii) involved in crucial organ-specific functions, and iv) expressed in a context of physiological inflammation. Evolutionary-determined adaptive immune responses against such non-CNS superautoantigens may provide a large range of functional benefits that do not relate to the CNS. As shown below, some of these may be species-specific (for instance, provide trophic support to organs that are essential to the survival and adaptation of a given species), while others may be shared between species (for instance, fighting against cancer cells).

*Species-restricted vs inter-species superautoantigens:* The advantages conferred to the host by an adaptive immune response directed against a superautoantigen may persist across evolution. In particular, one could anticipate that a substantial share of public TCRs are directed against two categories of superautoantigens: species-restricted and inter-species superautoantigens.

1) S *pecies-restricted superautoagntigens*: The choice of the term "species-restricted" refers to the notion that such antigens are not necessarily expressed in a species-specific manner. By contrast, the adaptive response mounted against these antigens is species-specific and confers a species-specific evolutionary advantage to the host. Notably, this may be the case for a group of brain-derived antigens that could have emerged as superautoantigens in *Homo sapiens*.

2) *Inter-species superautoantigens*: These antigens are not only expressed across distinct (or all) species endowed with an adaptive immune response, but the T-cell response mounted against these antigens confer an evolutionary advantage that is shared between such species.

*Natural antibodies directed against CNS antigens may exert only an indirect effect on neural repertoires:* Previous studies showed that CNS-directed antibodies can be detected in the blood in a large range of the healthy population ^186, 187^. However, antibodies do not or only poorly cross the blood-brain barrier, and cognition-promoting autoimmunity was demonstrated to rely on T-cells rather than autoantibodies ^54– 56, 188^. Thus, while T-cells and neural repertoires may be mutually supportive, only a one-way functional connection may link the antibody and neural repertoires. On the one hand, exposure of brain antigens to the immune system would benefit the host via an expansion/diversification of both the antibody and TCR repertoires, while on the other hand, only "self"-reactive T-cells (but not autoantibodies) would provide support to neural repertoires. Another explanation, not exclusive from the former one, would be that natural autoantibodies directed against CNS antigens participate in the afferent phase of T-cell mediated cognition-promoting immune responses. Engulfment of CNS-derived antigens opsonized or captured by secreted antibodies or, for B-cells, by membrane-bound immunoglobulins, may indeed result in antigen presentation to T-cells, notably in cervical lymph nodes.

*Natural autoantibodies and "self"-reactive T-cells may provide two separate arms of protective autoimmunity:* Although this point remains to be experimentally explored, one may anticipate that autoantibodies and "self"-reactive T-cells are targeting distinct (yet partially overlapping) groups of superautoantigens. T-cells and antibody repertoires would thus provide distinct and complementary facets of protective autoimmunity. One may suggest that for some (or possibly many) natural autoantibodies, an essential function is to somehow scavenge and buffer proteins that are renewed or exposed at a high rate in the blood. Supporting this view, spleen marginal zone B-cells, whose hosting tissue is directly plugged on the blood stream, are considered as a major source of natural autoantibodies ^189, 190^.

## Clinical implications

### Autoimmunity viewed as a neurodevelopmental disorder

The assumption that immune and neural repertoires are mutually supportive during developmental and post-developmental periods has potentially important clinical implications. In particular, if brain development, from the prenatal period to early adulthood, has a major impact on the acquisition and maintenance of immune repertoires, an endogenous neural origin of pathological autoimmunity may be envisioned. The window of time and immune context during which brain superautoantigens are initially exposed could be a major determinant of the diversity of the T-cell repertoire. In other words, a proper exposure to brain superautoantigens would determine the generation, maintenance and expansion of T-cells that not only recognize brain superautoantigens, but harbor a phenotype and functional profile that are ideally suited to support neural repertoires (i.e. *ad hoc* homing properties and *ad hoc* profiles of cytokines and neurotrophic factors). Similar principles may apply to B cells and natural autoantibodies, with the limitations discussed previously. 

Several elements of the literature support the notion that neural and immune repertoires mutually develop in humans. Cognitive and behavioral alterations are observed in children suffering from several categories of genetically-determined immunodeficiencies. These include severe combined immunodeficiencies ^191^ and the Di Georges syndrome, which associates thymic hypoplasia, cognitive deficits ^192^ and psychiatric manifestations, such as autism and schizophrenia ^193^. Conversely, complex immune alterations have been reported in patients suffering from the two most frequent neuropsychiatric and neurodevelopmental human disorders: autism and schizophrenia. Schizophrenia is associated with a higher incidence of autoimmune disorders, including Grave's disease, psoriasis and celiac disease ^194^. Moreover, a number of studies have demonstrated a large range of immune alterations in the blood of schizophrenic patients ^195, 196^. Also, the high incidence of autoimmune disorders in autistic patients and their siblings has suggested that autoimmune mechanisms may be involved in the pathophysiology of autism ^197^. However, in accordance with the co-development/co-evolution model, another explanation could be that the neurodevelopmental alterations characterizing autism and schizophrenia are the cause rather than the consequence of profound alterations of the immune repertoires, which may lead to pathological autoimmunity in a subgroup of these patients.

Interestingly, genome-wide association studies also provide important support to the co-development/co-evolution model. Indeed, as discussed earlier, this model predicts that genes involved in the diversification of both immune and neural repertoires have had a major evolutionary impact. Accordingly, there is now compelling evidence that genetic susceptibility to autism and schizophrenia is conferred, for some parts, by immune-related genes, including HLA genes ^198– 201^.

### Immune repertoires under the grip of the CNS

Besides periods of co-development and co-maturation, it is proposed that neural and immune repertoires mutually fuel each other during the whole life of an individual. Considering that CNS-derived antigens, similarly to microbiota-derived antigens, constantly shape immune repertoires, implies that mental state, learning tasks, cognitive activities and/or the execution of sensori-motor programs could exert major and specific effects on immune repertoires. Stress-induced alterations of the immune response is now extensively documented and is known to rely on two main pathways: the hypothalamus-pituitary-adrenal axis and the autonomic nervous system (ASN) pathway ^202, 203^. Recently, the brain reward system was also shown to deeply impact immune responses via signaling through the ASN ^204^. CNS-derived superautoantigens and their instructing roles on immune repertoires could thus provide another mechanism of brain-induced immunomodulation. More generally, demonstrating that neural and immune repertoires are functionally coupled could pave the way to innovative therapeutic strategies based on the control of adaptive immune responses by cognitive and/or sensorimotor tasks.

## Experimental insights

The assessment of immune repertoires by system biology approaches ^34, 36, 43, 205^ should allow the determination of whether or not cognitive activities, sensori-motor tasks and mental state directly impact immune repertoires. In particular, immune repertoires should be explored in experimental settings known to induce an increased synaptic plasticity in rodents (notably via enrichment of the environment). Similarly, murine genetic models of schizophrenia or autism should be investigated with regard to alterations of immune repertoires (this should be performed only if immune alterations are not expected to occur as a direct consequence of a given genetic manipulation). The same strategy could be also applied to murine models of mood disorders. Of note, recently-developed technologies, such as optogenetics and chemicogenetics, are currently being harnessed to unravel new links between the brain and immune system ^206^. Such innovative approaches should allow to precisely determine the impact exerted by the activation of specific synaptic circuits on: i) the peripheral T- and B-cell repertoires; and ii) the exposure of specific CNS antigens, notably via their draining to cervical lymph nodes. Finally, a global analysis of immune repertoires should be performed in human patients suffering from autism, schizophrenia or mood disorders. Notably, one may anticipate that qualitative or quantitative alterations of public TCRs may occur under these clinical conditions.
