# Supplementary material for: Evolution, immunity and the emergence of brain superautoantigens
Source: F1000Res. 2017 Feb 21;6:171. [Version 1] doi: 10.12688/f1000research.10950.1 (PMC5419255; doi:10.12688/f1000research.10950.1)
Supplement: Peer review history

# Peer review: Referee response for version 1

**DOI:** 10.5256/f1000research.11803.r22275

**Published:** 2017-05-09

**Authors:** Carmen Guaza

**Affiliations:** 1 Functional and Systems Neurobiology Department, Neuroimmunology Group, Cajal Institute, CSIC, Madrid, Spain

Nataf presents a sound and provocative opinion piece. The article gets readers to open their mind about the view that superautoantigens are shaped by physiological events associated with brain development and function, all in an integrative view. It is amazing the model of co-evolution of the immune and nervous system exposed in a brilliant and coherent way to promote open discussion that will better the field and impact clinical approaches.

I have read this submission. I believe that I have an appropriate level of expertise to confirm that it is of an acceptable scientific standard.

# Peer review: Referee response for version 1

**DOI:** 10.5256/f1000research.11803.r22001

**Published:** 2017-05-03

**Authors:** Abdelhadi Saoudi

**Affiliations:** 1 U1043, INSERM (French Institute of Health and Medical Research), Toulouse, France

This excellent opinion article by Serge Nataf is based on critical analysis of unrelated literature and proposes a sound model that bridge natural autoimmunity with brain development and functions. This model also raises several interesting biological questions (evolution, physiology and physiopathology) that will need the development of new experimental setting to understand the coevolution of the immune system and CNS.

I have read this submission. I believe that I have an appropriate level of expertise to confirm that it is of an acceptable scientific standard.

# Peer review: Referee response for version 1

**DOI:** 10.5256/f1000research.11803.r20397

**Published:** 2017-02-22

**Authors:** Irun R. Cohen

**Affiliations:** 1 Department of Immunology, Weizmann Institute of Science, Rehovot, Israel

This Opinion Article by Serge Nataf is a brilliant synthesis; it integrates seemingly unrelated observations to build a coherent model that explains unsung relationships between natural “homuncular” autoimmunity and nervous system development. Nataf’s model of the co-evolution of the immune and nervous systems is seminal biological thinking that bridges evolution, physiology, clinical medicine, health and disease. Readers will respond with challenges, experimental tests, and new understanding.

I have read this submission. I believe that I have an appropriate level of expertise to confirm that it is of an acceptable scientific standard.
